# Protopsyllidiidae from the Jiyuan Basin (Henan, China): Taxonomic Updates and Their Biostratigraphic Implications

**DOI:** 10.3390/insects17080824

**Published:** 2026-08-08

**Authors:** Marina Hakim, Minmin Xu, Diying Huang

**Affiliations:** 1 State Key Laboratory of Palaeobiology and Stratigraphy, Nanjing Institute of Geology and Palaeontology, Chinese Academy of Sciences, Nanjing 210008, China; marina@nigpas.ac.cn (M.H.); mmxu@nigpas.ac.cn (M.X.); 2 Department of Life and Earth Sciences, Faculty of Sciences II, Campus Pierre Gemayel, Lebanese University, Fanar, El-Matn 90656, Lebanon; 3 Nanjing College, University of Chinese Academy of Sciences, Nanjing 211135, China

**Keywords:** Hemiptera, Sternorrhyncha, Jurassic, Yanliao biota, Yangshuzhuang formation

## Abstract

Abundant new material of protopsyllidiids was collected from the late Middle Jurassic Yangshuzhuang Formation at Jiyuan City, Henan Province, Central China, and is described herein. The dominant form was assigned to the species “*Poljanka hirsuta* Yang, Yao & Ren, 2012”, first described from the Daohugou beds (Haifanggou Formation, Inner Mongolia, North China). Furthermore, the species is herein transferred from the genus *Poljanka* Klimaszewski, 1995 to *Cicadellopsis* Martynov, 1937 and renamed *Cicadellopsis hirsuta* (Yang, Yao & Ren, 2012) **comb. nov.** based on the presence of a forked vein RA in the forewings, as evidenced by the newly discovered specimens. A second protopsyllidiid species, *Poljanka jiyuanensis* **sp. nov.**, is also described and illustrated, showcasing the diversity of Protopsyllidioidea in the Mesozoic Chinese deposits. Abundant specimens of *Cicadellopsis hirsuta* **comb. nov.** have been discovered in both the Yangshuzhuang Formation at Jiyuan, Henan Province, and the Haifanggou Formation at Ningcheng, Inner Mongolia, further supporting their biostratigraphic correlation. This also indicates that the early assemblage of the Yanliao biota, represented by the Daohugou biota, was distributed at least as far south as Central China.

## Simple Summary

This work investigates fossil insects preserved in rock compressions from the Jurassic period in China. It aims to study records of protopsyllidiids, a family of extinct plant-feeding insects, to improve our understanding of their diversity, evolution, and biogeographic distribution. Firstly, a previously known species is transferred to a different genus based on newly observed wing features. Secondly, a newly discovered species is described and illustrated, revealing greater diversity of the protopsyllidiids during the Jurassic period. This insect family is recorded in the Henan Province of China for the first time, providing evidence that these ancient insect communities were more widely distributed than previously believed. These findings improve our knowledge of how insect groups evolved and spread through past environments, helping us to better understand the changes in biodiversity over geological time.

## 1. Introduction

The Middle-Late Jurassic Yanliao biota, alongside the Early Cretaceous Jehol biota, are known for their incredibly well-preserved diversity of abundant fossils. The records from these two famous Mesozoic terrestrial Lagerstätten of northeast China portray pivotal evolutionary events (e.g., transition towards flight and diversification of early bird and mammalian forms), as documented by their assemblages of invertebrates, fish, amphibians, salamanders, lizards, feathered dinosaurs, pterosaurs, early mammals, and plants [1,2,3,4,5,6,7,8].

The Yanliao biota encompasses various arthropods (insects, arachnids, and crustaceans), molluscs (bivalves and gastropods), vertebrates (fish, salamanders, lizards, pterosaurs, dinosaurs, and mammals), and vegetal remains (algae, fungi, plants, spores, and pollen) [4]. Insects are particularly abundant and very diverse in the Daohugou beds, as shown in [7], represented by Archaeognatha, Ephemeroptera, Odonata, Blattodea, Dermaptera, Embioptera, Grylloblattodea, Mantophasmatodea, Orthoptera, Phasmatodea, Plecoptera, Hemiptera, Permopsocida^†^, Lophioneuroptera^†^, Coleoptera, Diptera, Hymenoptera, Lepidoptera, Mecoptera, Megaloptera, Neuroptera, Raphidioptera, and Trichoptera.

One such insect group is the Protopsyllidioidea, an extinct hemipteran superfamily of the suborder Sternorrhyncha, discovered in abundance in the Yangshuzhuang Formation of the Jiyuan Basin and the uppermost layers of the Daohugou beds [6]. The superfamily is recorded worldwide, from Permian to Cretaceous, in deposits of both sedimentary rocks and amber (e.g., [9,10,11,12,13,14]).

**Figure 1:**
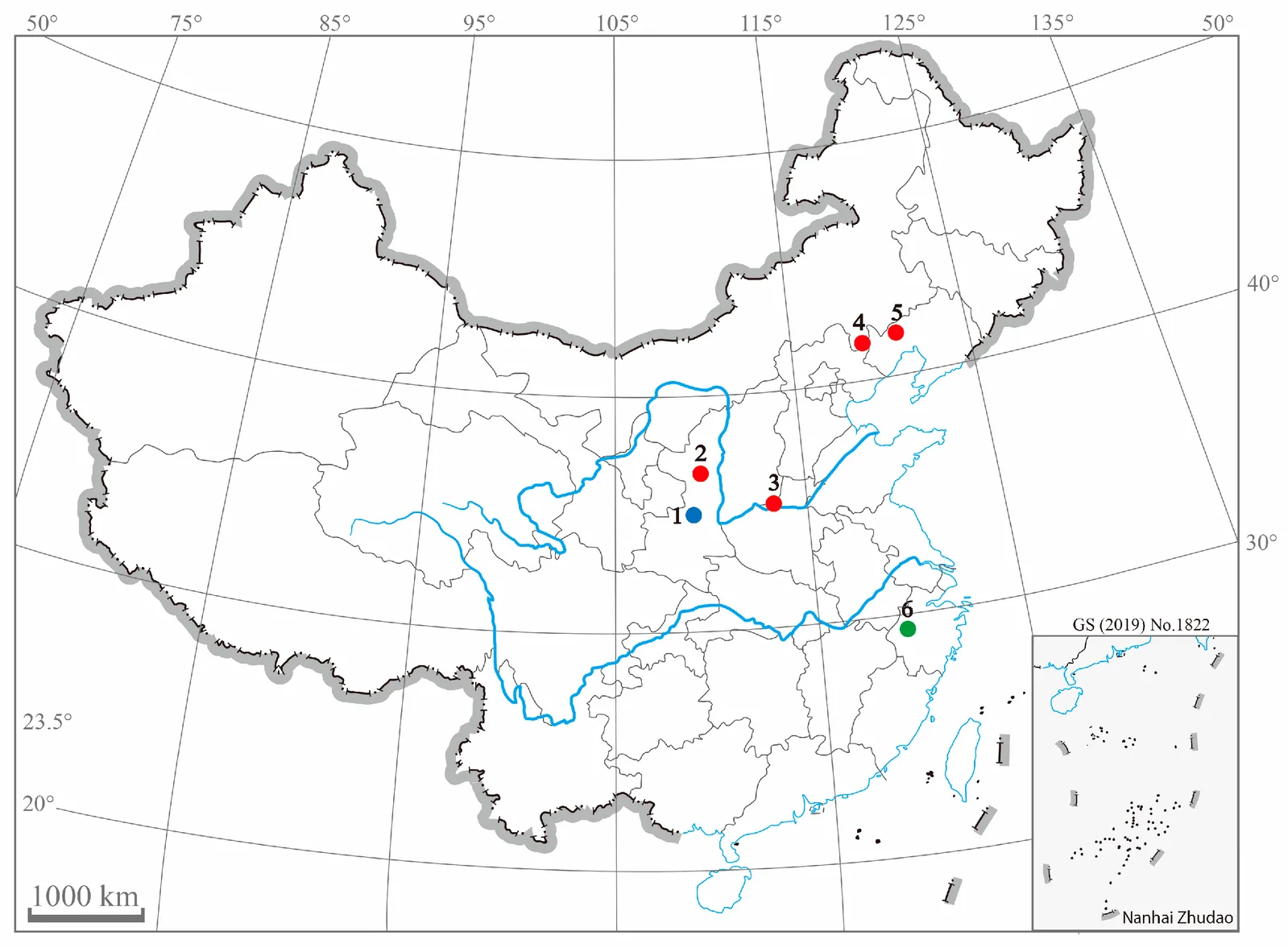
Distribution map of protopsyllidioid records in Chinese Mesozoic outcrops: 1. Tongchuan Formation (Shaanxi); 2. Yan’an Formation (Shaanxi); 3. Yangshuzhuang Formation (Henan); 4. Haifanggou Formation (Inner Mongolia, Daohugou area); 5. Haifanggou Formation (Liaoning); 6. Shouchang Formation (Zhejiang). Coloured dots indicate the following: blue dot, Triassic; red dot, Jurassic; green dot, Cretaceous.

Protopsyllidioids are frequent in Mesozoic Chinese deposits, particularly in the Jurassic strata of North China (Table 1 and Figure 1). To date, only one representative is known from the South of China, viz. the Cretaceous species *Paraprotopsyllidium shouchangense* Hakim & Huang, 2023 from Zhejiang Province [15]. It is noteworthy that Hakim & Huang (2024) also described a possible protopsyllidioid, namely *Fujianella paradoxa* Hakim & Huang, 2024 from Fujian Province, Southeast China, of Early Cretaceous age, which they placed in the Sternorrhyncha, Hemiptera [16].

A total of nine species belonging to six genera have currently been discovered in Chinese deposits, systematically described, and assigned to the superfamily (Table 1). Nonetheless, before this work, no representatives had yet been described from the Yangshuzhuang Formation, despite some specimens having been illustrated by J.F. Zhang in [17].

**Table 1:** List of protopsyllidioid taxa from Chinese deposits.

Species	Family	Type Formation and Locality	Other Locality	Age
*Cicadellopsis hirsuta* (Yang, Yao & Ren, 2012) **comb. nov.** [18]	Protopsyllidiidae	Haifanggou Formation, Inner Mongolia	Yangshuzhuang Formation, Henan Province (**herein**)	Middle Jurassic
*Paraprotopsyllidium shouchangense* Hakim & Huang, 2023 [15]	Paraprotopsyllidiidae	Shouchang Formation, Zhejiang Province		Early Cretaceous (Barremian)
*Poljanka curticapillata* Lü, Luo & Yao, 2023 [12]	Protopsyllidiidae	Haifanggou Formation, Inner Mongolia		Middle Jurassic
*Poljanka jiyuanensis* **sp. nov.**	Protopsyllidiidae	Yangshuzhuang Formation, Henan Province (**herein**)		Middle Jurassic
*Poljanka strigosa* Yang, Yao & Ren, 2013 [19]	Protopsyllidiidae	Haifanggou Formation, Inner Mongolia		Middle Jurassic
*Sinopsocus oligovenus* Lin, 1976 [20]	Protopsyllidiidae	Haifanggou Formation, West Liaoning Province		Middle Jurassic
*Sinopsocus yananensis* Xu, Hakim & Huang, 2023 [13]	Protopsyllidiidae	Yan’an Formation, Shaanxi Province		Middle Jurassic
*Subaphidulum sinica* Xu, Hakim & Huang, 2023 [13]	Protopsyllidiidae	Yan’an Formation, Shaanxi Province		Middle Jurassic
*Triassopsyllidiida pectinata* Huang, Hakim, Fu & Nel, 2022 [21]	Permopsyllidiidae?	Yanchang Formation, Shaanxi Province		Middle–Late Triassic

The genus *Cicadellopsis* was initially established by Martynov, 1937 as a Homoptera *incertae sedis* based on the type species *Cicadellopsis incerta* Martynov, 1937 [22]. Evans (1956) later stated that the wing is psyllid in type and placed it in the family Protopsyllidiidae [23]. Becker-Migdisova (1985), in their extensive work on protopsyllidiids, presented a generic diagnosis for *Cicadellopsis* after describing an additional five species from Mesozoic rock compressions [9]. Klimaszewski (1995) then established the genus *Poljanka* [24] to include four of the five species added to *Cicadellopsis* by [9]. Another three new species were later assigned to *Poljanka* by [12,18,19]. Following these works, the genus *Cicadellopsis* now englobes two species: *Cicadellopsis incerta* from the Early Jurassic, Kyzyl-Kiya, Tadzhikistan, and *Cicadellopsis issykkulica* Becker-Migdisova, 1985 from the Late Triassic, Issyk-Kul, Kirghizstan. Meanwhile, the genus *Poljanka* englobes seven species (+two unnamed species; refer to [9,24]): *Poljanka shurabensis* (Becker-Migdisova, 1985) from the Middle Jurassic, Shurab, Tadzhikstan; *Poljanka kukalovae* (Becker-Migdisova, 1985), *Poljanka sharovi* (Becker-Migdisova, 1985), and *Poljanka ventriculosa* (Becker-Migdisova, 1985) from the Late Jurassic, Karatau, Kazachstan; *Poljanka curticapillata*, “*Poljanka hirsuta*”, and *Poljanka strigosa* from the Middle Jurassic, Daohudou beds, China.

In the present article, we establish the species *Cicadellopsis hirsuta* **comb. nov.** based on newly discovered material and describe a new species of *Poljanka*. Both species of Protopsyllidiidae documented herein originate from the Jiyuan basin (Yangshuzhuang Formation), and thus, we discuss the co-occurrence of this insect group in the Daohugou beds.

## 2. Material and Methods

The study material was preserved in the grey–green mudstone layers of the uppermost sections of the Yangshuzhuang Formation, believed to be late-Middle Jurassic in age, near Anyao Village, Jiyuan City, Henan Province, Central China. It consists of 48 specimens assigned to *Cicadellopsis* Martynov, 1937 and four specimens to *Poljanka* Klimaszewski, 1995; all material is deposited at the Nanjing Institute of Geology and Palaeontology, CAS, China.

Some specimens were carefully prepared by removing the overlying rock matrix using a sharp knife. The fossils were photographed using a digital camera attached to a Zeiss Discovery V16 stereomicroscope, and stacked using Helicon Focus 8 software. The plates, including the line drawings, were processed and enhanced using Adobe Photoshop CC 2019 graphic software.

We follow the wing venation nomenclature of [25]. The wing venation nomenclature abbreviations are as follows: A, anal vein; CuA, cubitus anterior; CuP, cubitus posterior; M, media; RA, radius anterior; RP, radius posterior. For *Cicadellopsis hirsuta* **comb. nov.**, measurements of the forewing are based on specimen NIGP210462 and measurements of the hind wing are based on specimen NIGP210488. For *Poljanka jiyuanensis* **sp. nov.**, measurements of the forewing are based on the holotype specimen NIGP210481. All wings are measured along the midline from the base to the apex.

This article is registered in ZooBank with the following LSID (reference): urn:lsid:zoobank.org:pub:F2A6B48B-AF7B-44DC-BD15-EB7D14E8F3E3.

### Geological Setting

**Figure 2:**
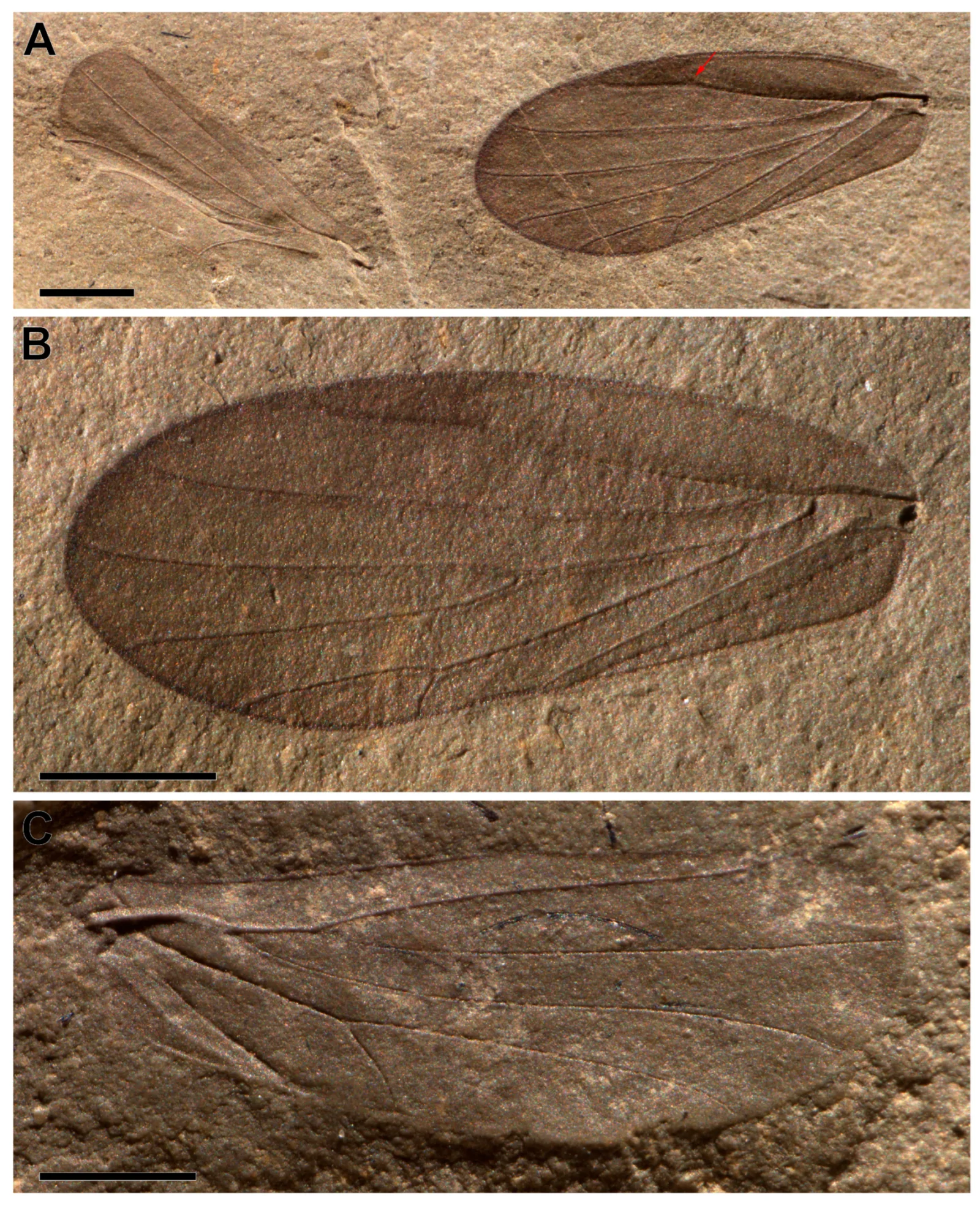
*Cicadellopsis hirsuta* (Yang, Yao & Ren, 2012) **comb. nov.**: (**A**) specimen NIGP210462; bifurcation of RA indicated by red arrow; (**B**) forewing, specimen NIGP210462; (**C**) hind wing, specimen NIGP210488. Scale bars = 1 mm.

**Figure 3:**
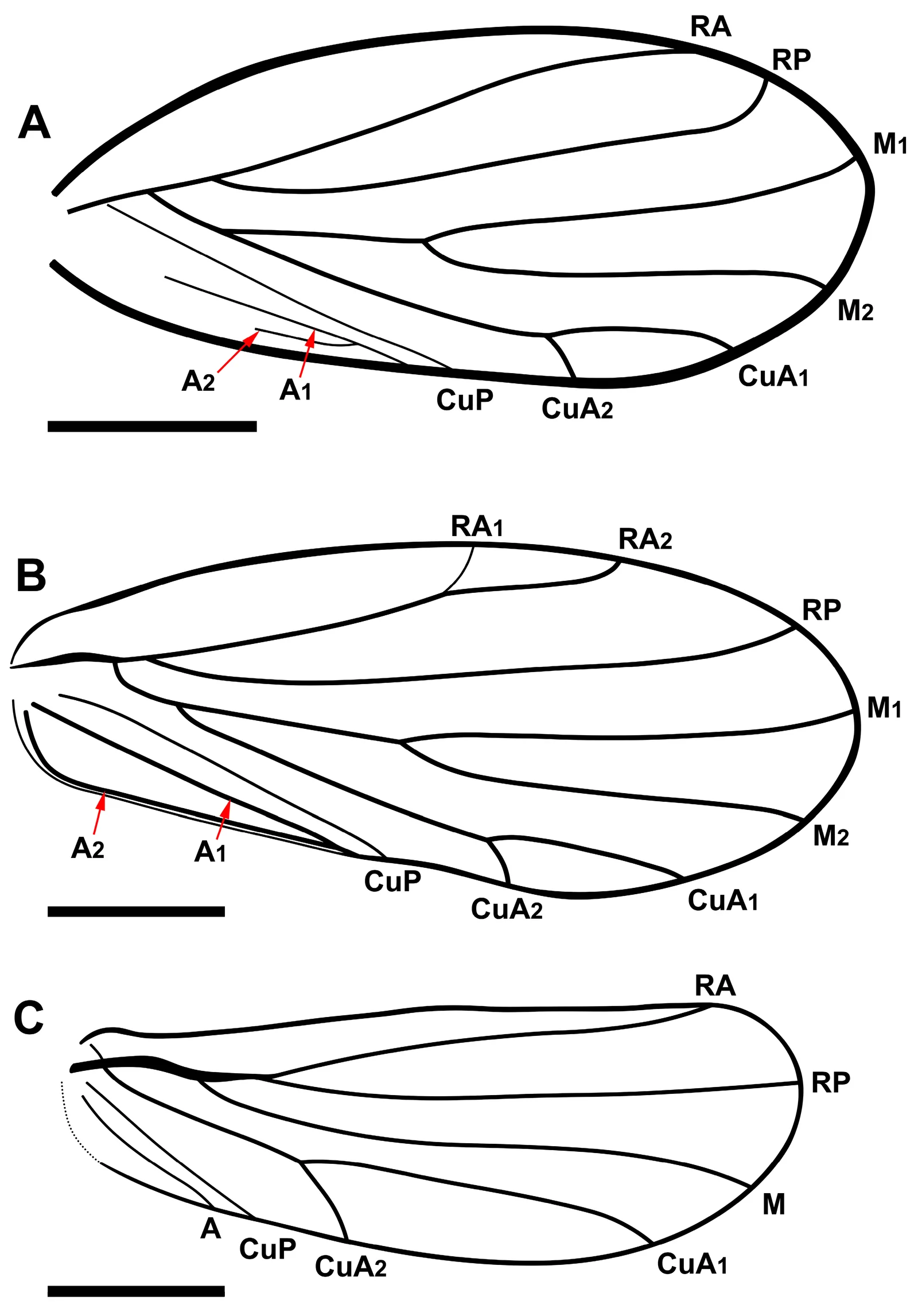
*Cicadellopsis hirsuta* (Yang, Yao & Ren, 2012) **comb. nov.**: (**A**) original illustration of forewing by Yang et al. (2012); (**B**) hand drawing of forewing based on NIGP210462; (**C**) drawing of hind wing based on NIGP210488. Scale bars = 1 mm.

The Jiyuan Basin is located in northwestern Henan Province, at the southwestern margin of the North China Craton (also referred to as the North China Block or Basin), and southeast of the Ordos Basin [26] (figure 1), [27] (figures 1 and 6), [28] (figure 1). Geographically, it is generally confined to the area west of the Kaifeng Depression and exhibits an overall east–west structural orientation. The basin lies adjacent to the southern margin of the Qinling Orogenic Belt and connects tectonically with the Taihang Mountains. Its eastern portion was significantly influenced by the uplift of the North China Block during the late Middle Triassic, which triggered intense tectonic activity in the region [29]. The Yangshuzhuang Formation of the Jiyuan Basin, dated back to the Middle Jurassic, overlays the Anyao Formation and underlays the Ma’ao Formation through unconformity [6] (figure 8), [26] (figures 2 and 3), [30,31]. The insect-bearing layers are mainly composed of grey–greenish mudstone. The study material was collected from such layers in the uppermost sections of the Yangshuzhuang Formation, near Anyao Village, Chengliu Township, Jiyuan City, Henan Province, China [6] (figure 7D).

The insect record described from this formation is considered Middle Jurassic (possibly Callovian) in age [13]; fossils are abundant and represented to date by four reported orders, i.e., Diptera, Hemiptera, Mecoptera, and Odonata [6,13,32,33,34,35,36,37,38,39,40]. Huang et al. (2018) stated that protopsyllididioids are dominantly plentiful in the Yangshuzhuang Formation, similarly to the top layers of the Daohugou beds [6]. In fact, a biostratigraphic correlation was proposed between the Yangshuzhuang Formation and the Daohugou beds based on conchostracan assemblages [6,41].

## 3. Results

### Systematic Palaeontology

**Figure 4:**
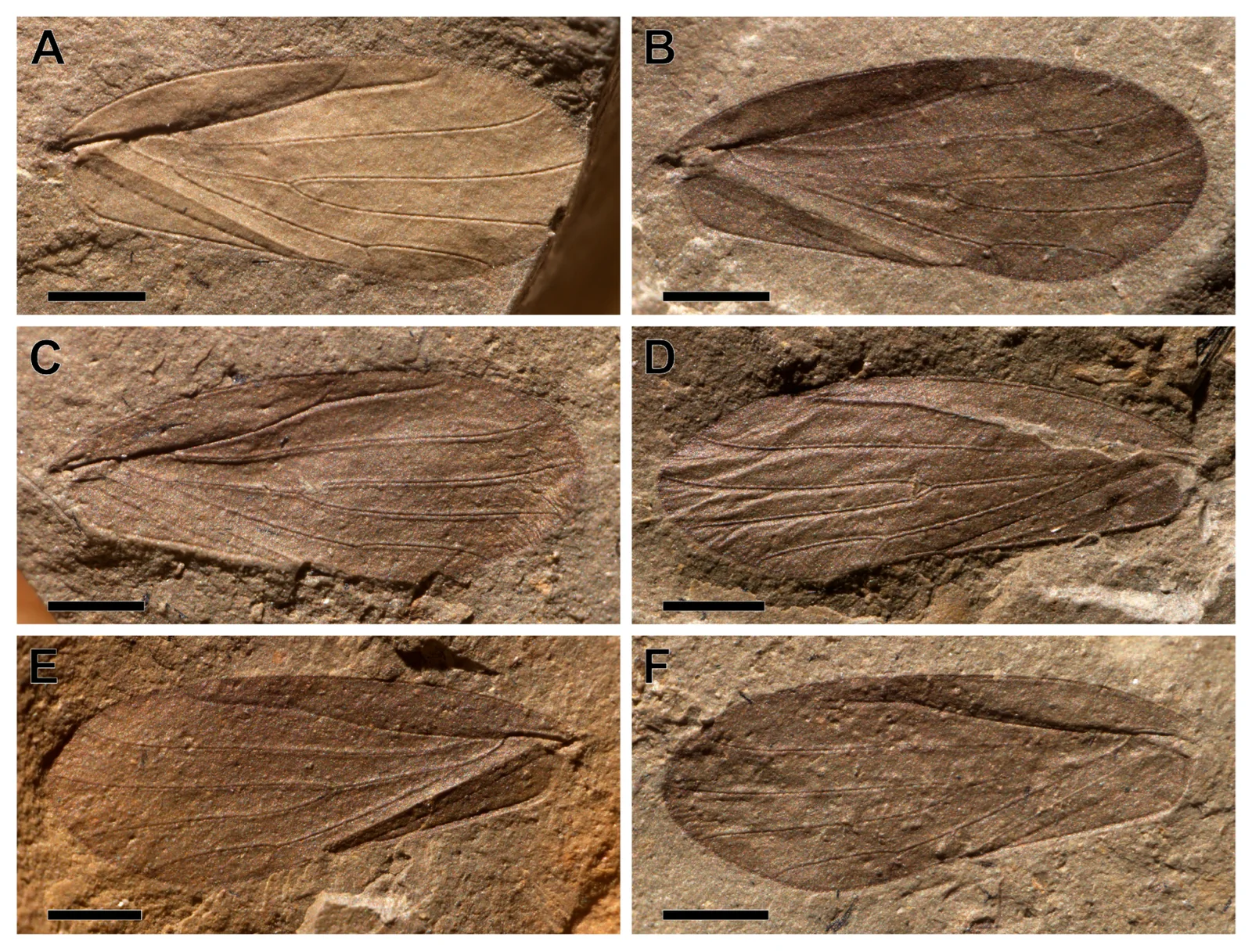
*Cicadellopsis hirsuta* (Yang, Yao & Ren, 2012) **comb. nov.**, forewing: (**A**) specimen NIGP210463; (**B**) specimen NIGP210464; (**C**) specimen NIGP210465; (**D**) specimen NIGP210466; (**E**) specimen NIGP210467; (**F**) specimen NIGP210468b. Scale bars = 1 mm.

**Figure 5:**
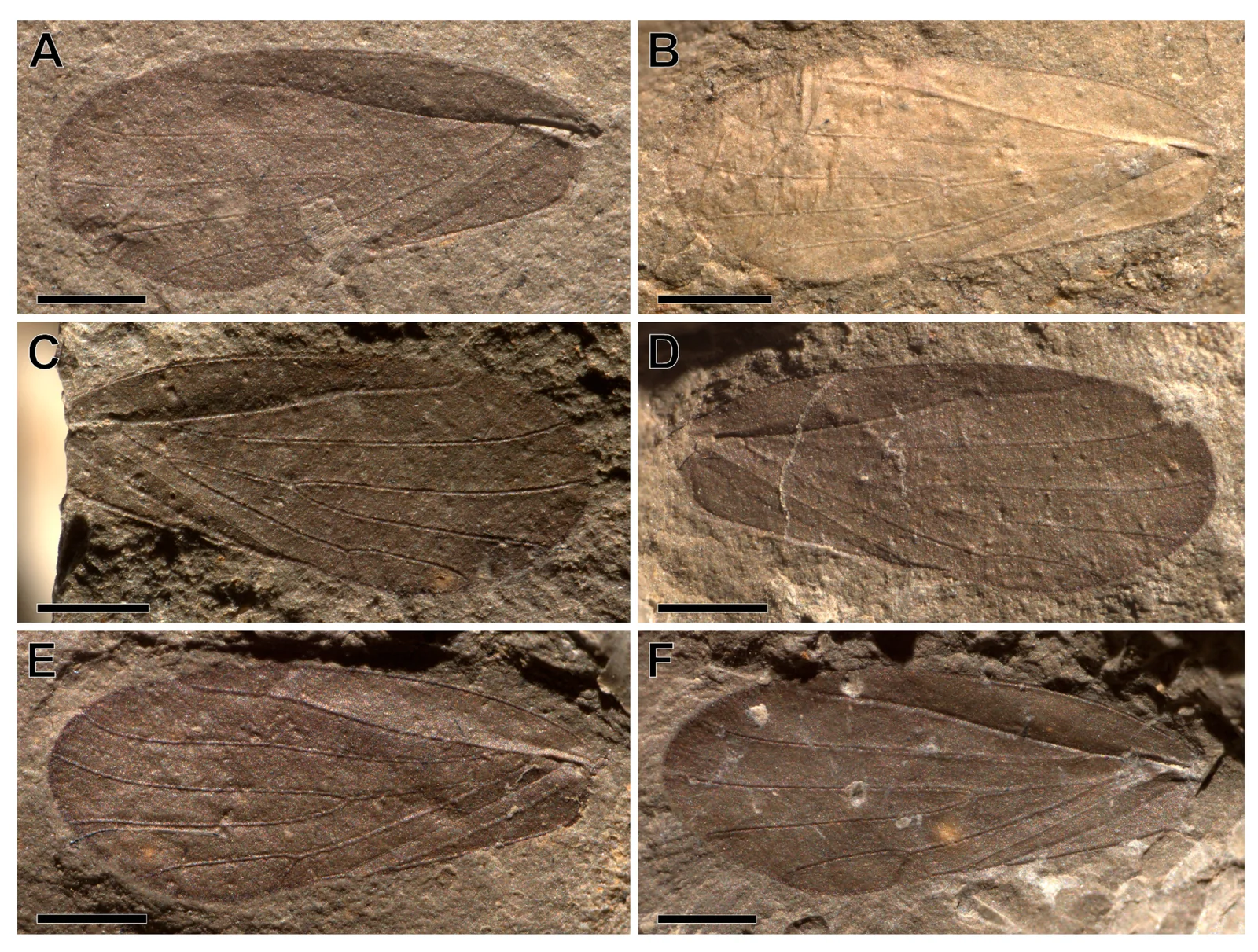
*Cicadellopsis hirsuta* (Yang, Yao & Ren, 2012) **comb. nov.**, forewing: (**A**) specimen NIGP210475; (**B**) specimen NIGP210476; (**C**) specimen NIGP210477; (**D**) specimen NIGP210478; (**E**) specimen NIGP210479; (**F**) specimen NIGP210480. Scale bars = 1 mm.

**Figure 6:**
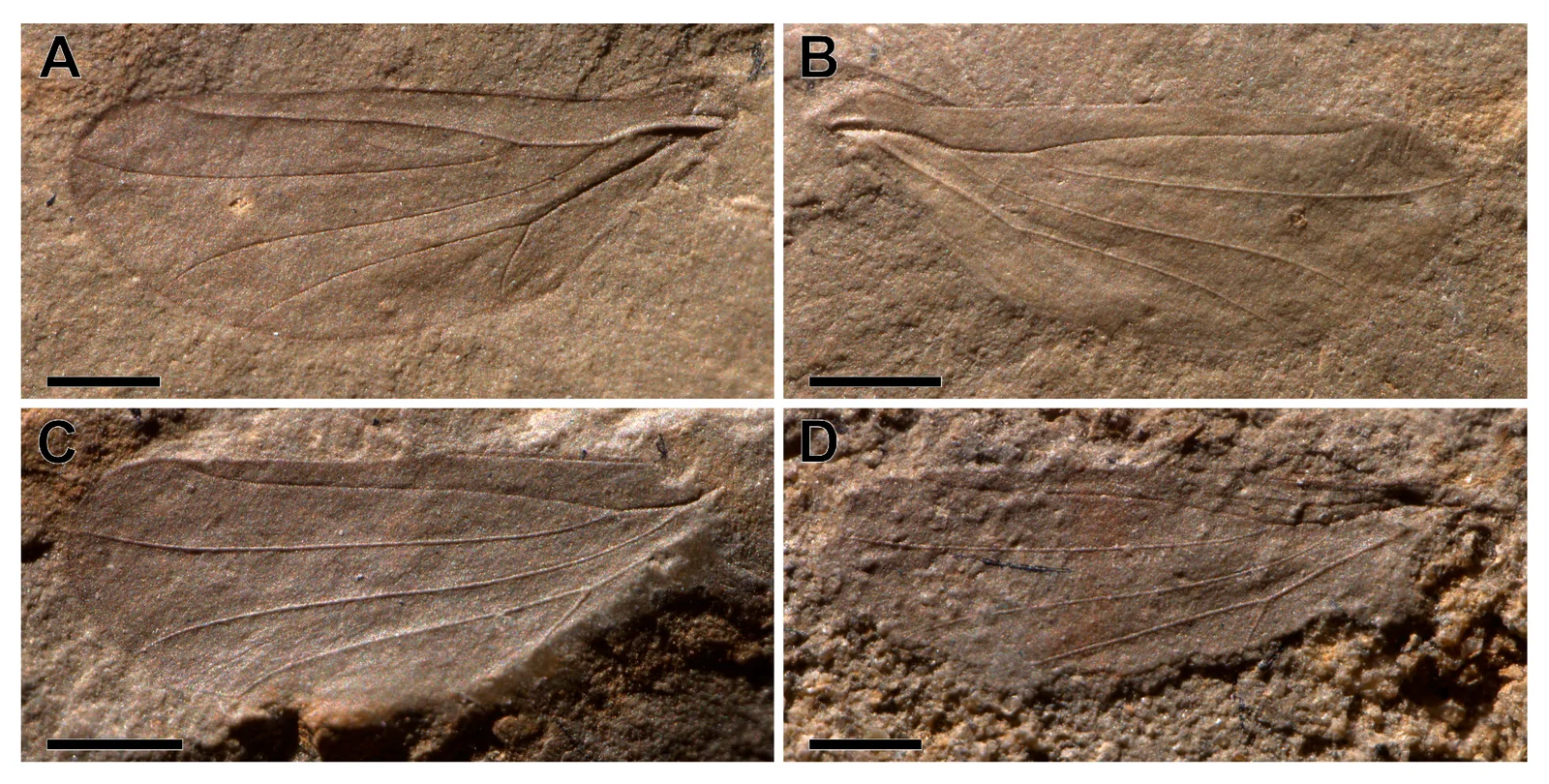
*Cicadellopsis hirsuta* (Yang, Yao & Ren, 2012) **comb. nov.**, hind wing: (**A**,**B**) specimen NIGP210485 (part and counterpart); (**C**) specimen NIGP210486; (**D**) specimen NIGP210487. Scale bars = 1 mm.

**Figure 7:**
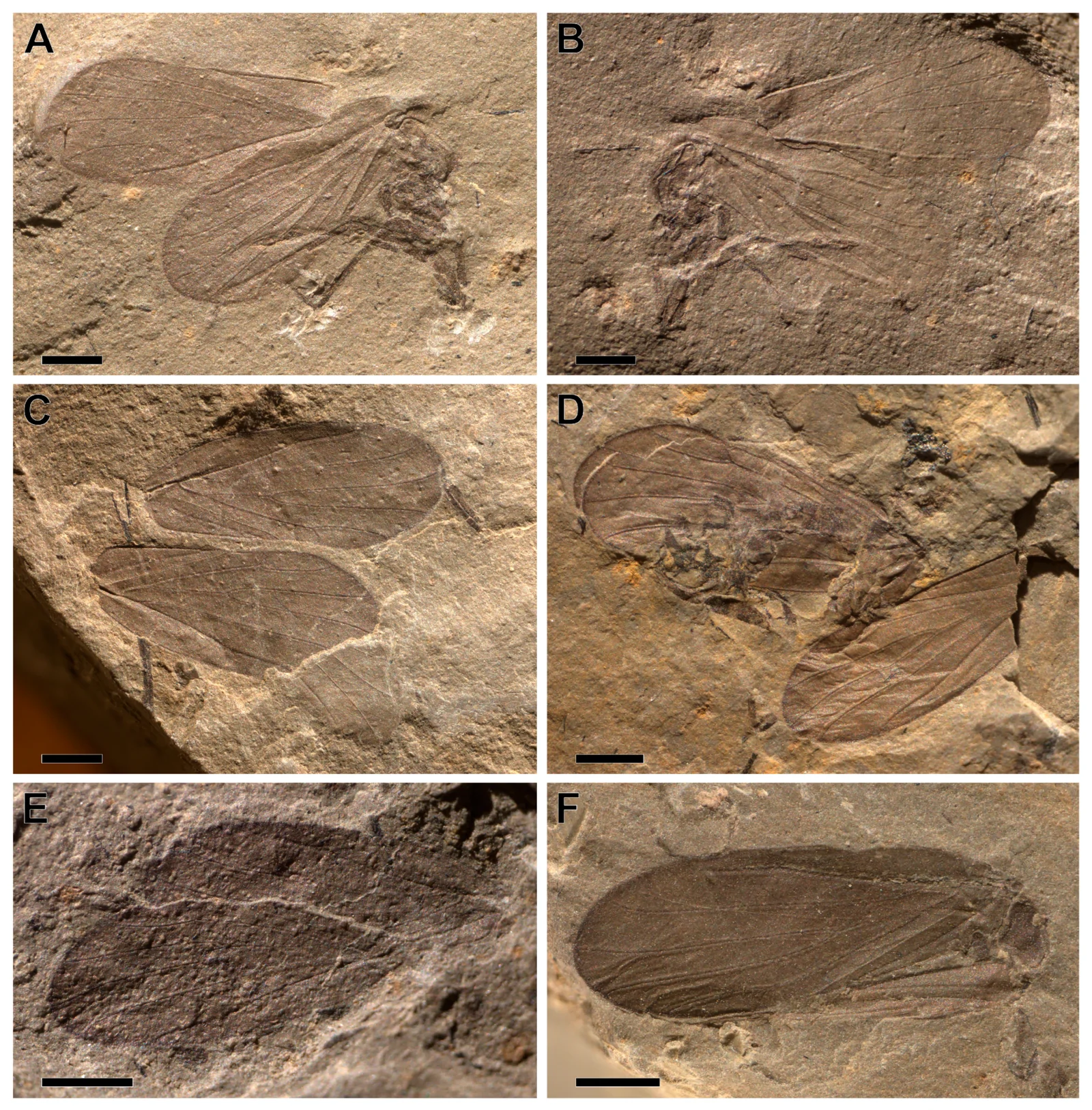
*Cicadellopsis hirsuta* (Yang, Yao & Ren, 2012) **comb. nov.**: (**A**,**B**) specimen NIGP210470 (part and counterpart); (**C**) specimen NIGP210471; (**D**) specimen NIGP210472; (**E**) specimen NIGP210473; (**F**) specimen NIGP210474. Scale bars = 1 mm.

Order Hemiptera Linnaeus, 1758 [42]
Suborder Sternorrhyncha Amyot & Audinet-Serville, 1843 [43]
Superfamily Protopsyllidioidea Carpenter, 1931 [44]
Family Protopsyllidiidae Carpenter, 1931 [44]
Genus *Cicadellopsis* Martynov, 1937 [22]
Type species. *Cicadellopsis incerta* Martynov, 1937 [22]
*Cicadellopsis hirsuta* (Yang, Yao & Ren, 2012) **comb. nov.**

(Figure 2, Figure 3, Figure 4, Figure 5, Figure 6 and Figure 7)

**Additional material.** NIGP210462–NIGP210468, NIGP210470–NIGP210480, NIGP210485–NIGP210502, and NIGP210610–NIGP210621; all specimens deposited at the Nanjing Institute of Geology and Palaeontology, Chinese Academy of Sciences.

**Locality and horizon.** Type material: Haifanggou Formation, Daohugou Village, Ningcheng County, Inner Mongolia, China; Middle Jurassic. New material: Yangshuzhuang Formation, Anyao Village, Chengliu Township, Jiyuan City, Henan Province, China; Middle Jurassic.

**Updated diagnosis.** After the original diagnosis of [18], the following adjustments were made: forewing length to width ratio of 2.2–2.5:1; RA forked with RA_1_ faint; fork M + CuA shortly after fork R, stem M + CuA 1.3–1.6 times the length of stem R; fork M_1_ + M_2_ around 1/2 the wing length, longer than common stem M and between 1.6 and 2.6 times its length; CuA cell small, around 1/4 of the wing length, length to width ratio of CuA cell 3–3.8:1. Hind wing length to width ratio of 2.6–2.9:1; no cross-veins; elongate fork R at basal 1/3 of wing length, elongate CuA cell slightly less than 1/2 (around 45%) the wing length, branches of CuA longer than common stem CuA; anal region small with one anal vein.

**Remarks.** The new material mainly consists of well-preserved forewings and hind wings; thus, in the section above, we revise the diagnosis regarding these two body structures only. The original diagnosis by [18] also includes characters based on proportions of some antennal and legs structures.

Moreover, vein RA_1_ is very faint; it is clearly delimited in some specimens (Figure 4A–C) while not visible in others (Figure 4D,F). Nonetheless, vein RA always displays obtuse angular-like bending near the suspected level of branching into RA_1_ and RA_2_, regardless of the status of RA_1_ in the preserved forewings. We also note some variability in the length of stem M compared to the length of fork M_1_ + M_2_ among some individuals, but it is always shorter than the fork.

**Description.** Forewing elongate, length 4.8 mm (range of 4.6–5.6 mm in other individuals) and maximum width 2.0 mm (range of 1.9–2.3 mm in other individuals), length to width ratio 2.4:1; all veins except CuP setose, indicated by seta insertion points; costa curved, costal cell broad, widest around middle part; common stem R + M + CuA branching into veins R and M + CuA at 1/9 (11%) of wing length; common stem R short (5% of wing length), branching into RA and RP around 1/7 (14%) of wing length; RA forked, RA_1_ short, straight and faint, RA_2_ weakly curved, 3 times the length of RA_1_; RP simple, elongate, weakly sinusoidal, apically directed towards RA; common stem M + CuA short (8% of wing length), slightly longer than common stem R, branching shortly after R into veins M and CuA at 1/5 (20%) of wing length; common stem M shorter than branches M_1_ and M_2_, about 1/2 the length of M_1_; M cell elongate and narrow, widening distally; M_1_ and M_2_ diverging apically; common stem CuA elongate, longer than fork CuA_1_ and CuA_2_, about 3/2 the length of CuA_1_; CuA_1_ weakly curved, semi-straight in mid-section, CuA_2_ distinctly shorter than CuA_1_, about 1/4 of its length; CuA cell semi-triangular, elongate, ratio length to width 3.8:1; CuP simple, straight; A_1_ straight, A_2_ weakly curved and very close to basal posterior wing margin; anal veins joining shortly before reaching margin, forming a long Y-shape. Hind wing elongate, length 4.3 mm (range of 3.7–4.3 mm in other individuals) and maximum width 1.5 mm (range of 1.4–1.5 mm in other individuals), ratio length to width 2.9:1; CuA emerging near wing base; common stem R + M present, and longer than common stem R; R forked into elongate RA and RP around 1/5 of wing length, RA weakly sinusoidal, RP semi-straight; no cross-veins present; M, CuP and A simple; M sinusoidal; CuA forked, common stem CuA 2/3 the length of CuA_1_; CuP straight; anal region small, no second anal vein.

**Figure 8:**
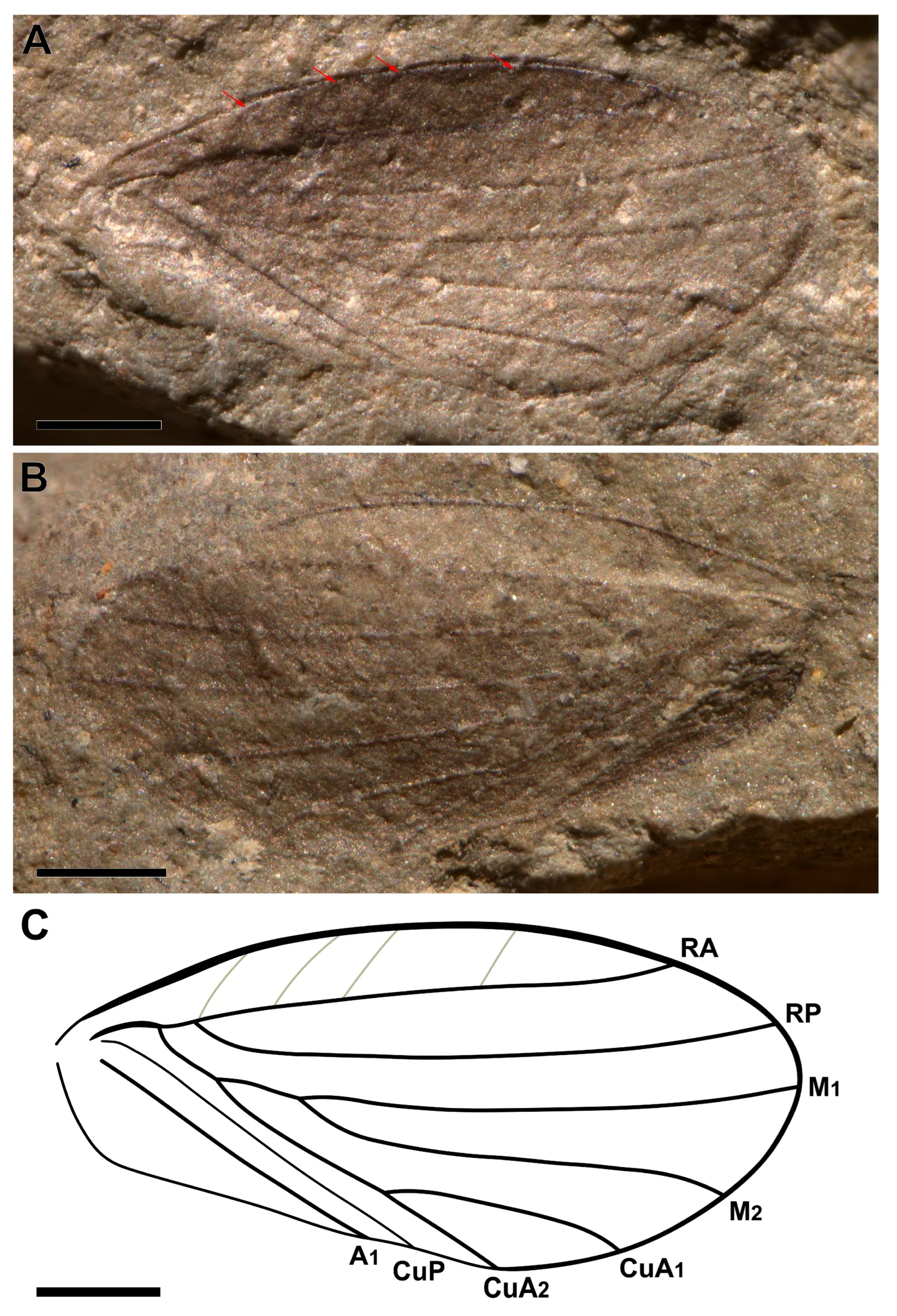
*Poljanka jiyuanensis* **sp. nov.**, holotype, specimen NIGP210481: (**A**,**B**) forewing (part and counterpart), potential veinlets indicated by red arrows; (**C**) hand drawing. Scale bars = 0.5 mm.

**Figure 9:**
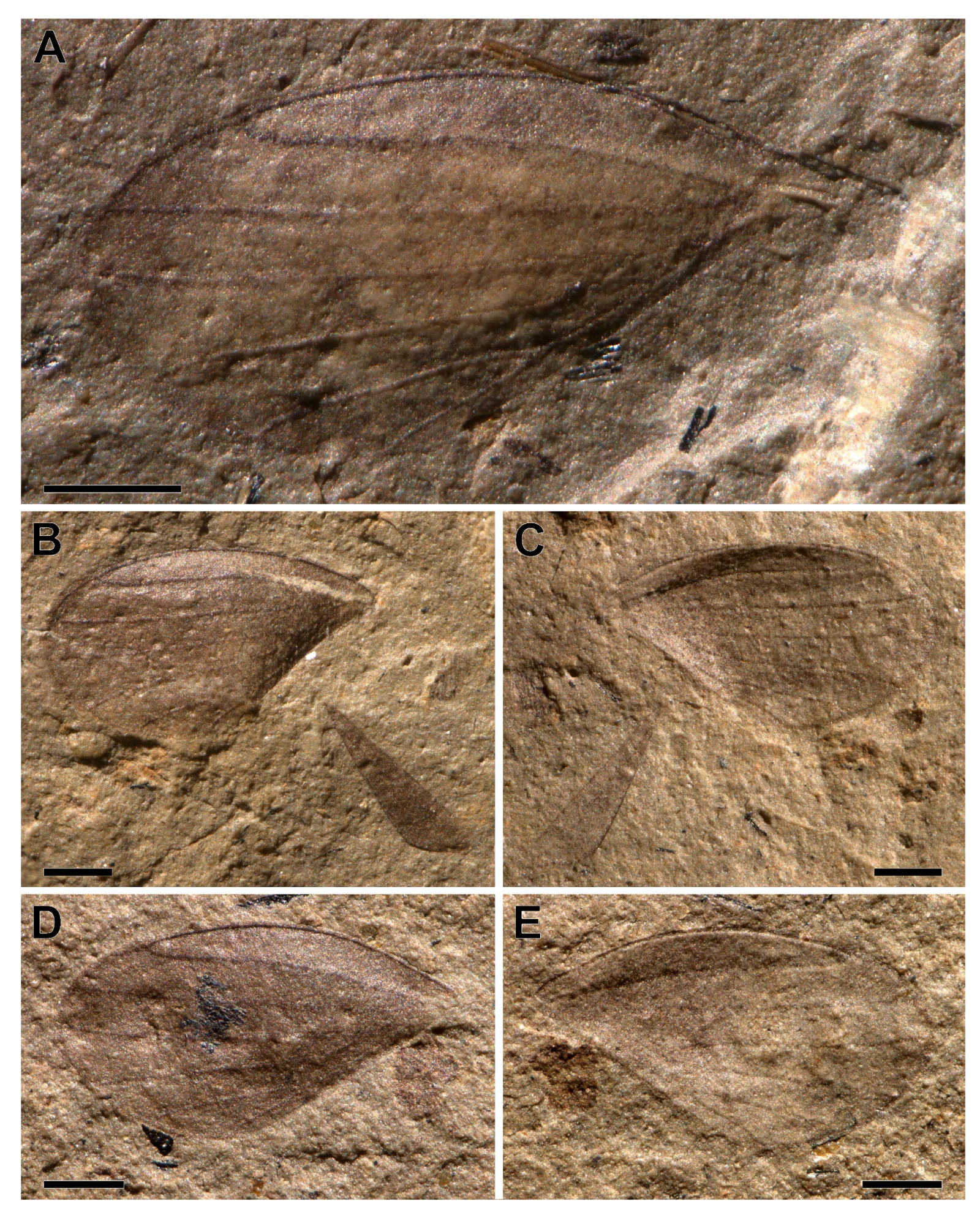
*Poljanka jiyuanensis* **sp. nov.**, forewing: (**A**) specimen NIGP210482; (**B**,**C**) specimen NIGP210483 (part and counterpart); (**D**,**E**) specimen NIGP210484 (part and counterpart). Scale bars = 0.5 mm.

**Figure 10:**
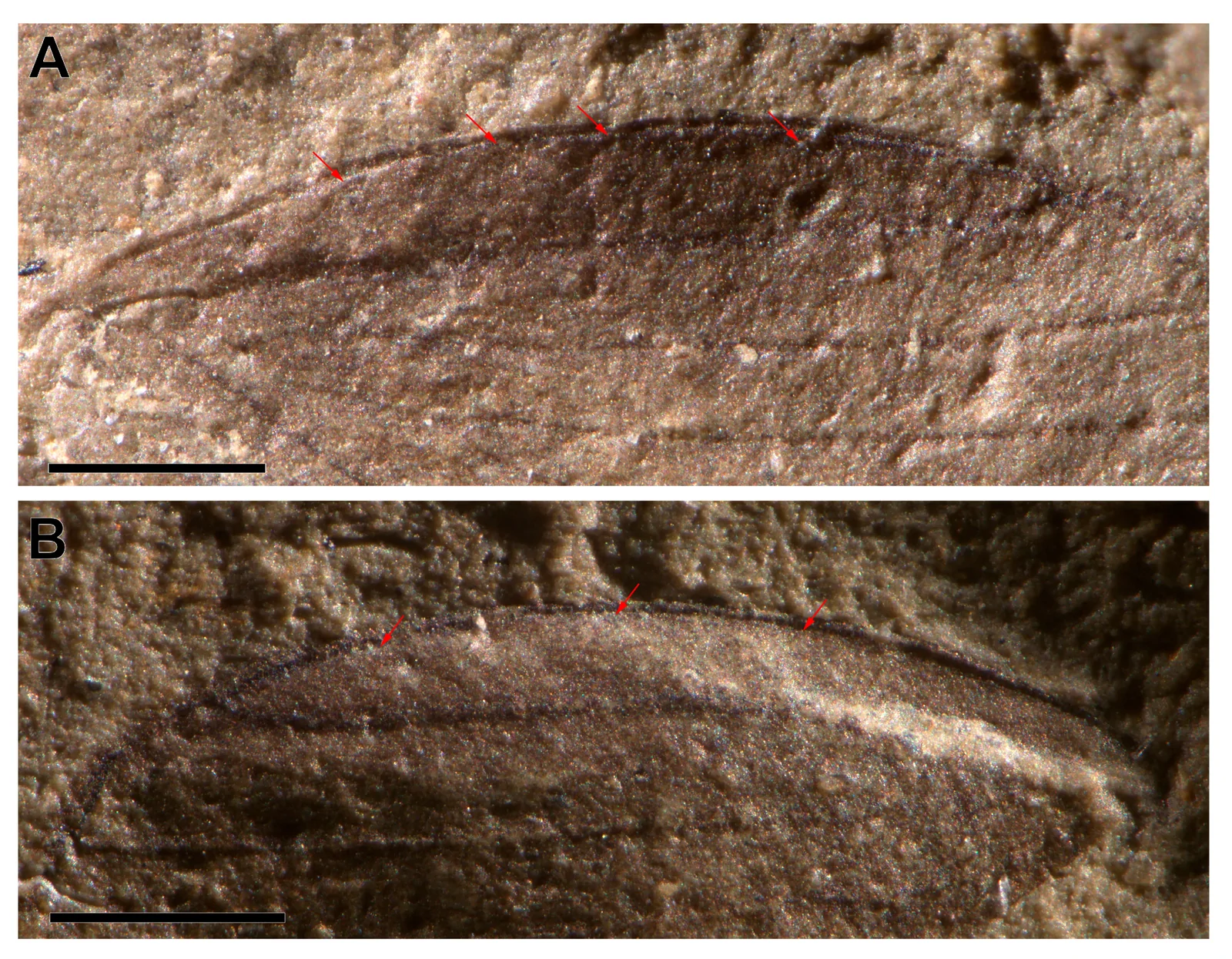
*Poljanka jiyuanensis* **sp. nov.**, potential veinlets (indicated by red arrows) in forewing: (**A**) holotype, specimen NIGP210481; (**B**) paratype, specimen NIGP210483b. Scale bars = 0.5 mm.

Genus *Poljanka* Klimaszewski, 1995 [24]
Type species. *Poljanka shurabensis* (Becker-Migdisova, 1985) [9]
*Poljanka jiyuanensis* **sp. nov.**
(Figure 8, Figure 9 and Figure 10)
LSID. urn:lsid:zoobank.org:act:FCE96C91-5A9F-4B14-86C0-F1A3C8D68448

**Type material.** Holotype specimen NIGP210481 (a, b); paratype specimens NIGP210482, NIGP210483 (a, b), and NIGP210484 (a, b); all specimens deposited at the Nanjing Institute of Geology and Palaeontology, Chinese Academy of Sciences.

**Etymology.** The specific epithet refers to the Jiyuan basin, the locality where the specimens were collected.

**Locality and horizon.** Yangshuzhuang Formation, near Anyao Village, Chengliu Township, Jiyuan City, Henan Province, China; Middle Jurassic.

**Diagnosis.** Forewing length to width ratio 1.8–2:1; Sc absent; stem R shorter than M + CuA (about half the length), stem R branching near wing base; stem M shorter than fork (about 1/6 the length of M_1_), branching at 1/3 of wing length; CuA cell semi-triangular, with sharp edges; CuA_2_ slightly over half the length of CuA_1_.

**Description.** Forewing broad, length 2.9 mm (range of 2.4–2.9 mm in other individuals) and maximum width 1.4 mm (similar in other individuals), ratio length to width 1.8–2:1; all veins except CuP setose, indicated by seta insertion points; costa curved; common stem R + M + CuA branching into veins R and M + CuA around 1/7 (13%) of wing length; common stem R short (5% of wing length), branching into elongate RA and RP at 1/5 (20%) of wing length; RA simple, semi-straight, potential weak veinlets emerging from RA and reaching wing margin; RP simple, curved, apically directed towards RA; common stem M + CuA short (10% of wing length), longer than common stem R, branching shortly after R into veins M and CuA around 1/5 (22%) of wing length; common stem M much shorter than branches M_1_ and M_2_, about 1/6 the length of M_1_; M cell very elongate, narrow at base and widening distally; M_1_ and M_2_ diverging apically; common stem CuA shorter than fork CuA_1_ and CuA_2_, about 4/5 the length of CuA_1_; CuA_1_ weakly curved, semi-straight in mid-section, CuA_2_ shorter than CuA_1_, a little over 1/2 of its length; CuA cell semi-triangular, elongate, ratio length to width 3.5:1; A_1_ straight, A_2_ not visible, possibly running very close to wing margin.

## 4. Discussion

Klimaszewski (1995) distinguished *Poljanka* from *Cicadellopsis* based on the following characters of the forewing: (1) vein RA simple (instead of bifurcated), (2) vein R shorter than M + CuA (instead of longer), and (3) longer anal field [24]. However, we note that, in [9], the stem R is also depicted as shorter in length than M + CuA in both of the species *C. incerta* and *C. issykkulica*. Meanwhile, stem R is subequal to M + CuA in *P. sharovi*, longer in *P. curticapillata*, and unknown in *P. kukalovae* or *P. shurabensis* [9,12]. Consequently, the diagnostic character [stem R shorter/longer than M + CuA] used by [24] to differentiate between the two genera appears unsuitable. On the other hand, the length of the anal field does not seem significantly different between *P. strigosa*, *C. hirsuta* **comb. nov.** and *C. incerta*.

*Cicadellopsis hirsuta* **comb. nov.** was initially described from two well-preserved individuals, collected from the Daohugou beds, and attributed to *Poljanka* following the revised diagnosis of the genus by [18]. They discussed that *Cicadellopsis hirsuta* **comb. nov.** would fall into *Poljanka* based on the following characters: (1) swollen apical flagellomere, (2) first tarsomere longer than second tarsomere, and (3) forewing with RA_1_ reduced, vein R slightly shorter than M + CuA, and a bifurcated CuA. Firstly, the trait [stem R shorter/longer than M + CuA]—as discussed previously—is unreliable for comparing *Cicadellopsis* and *Poljanka*. Secondly, the character [CuA two-branched] is found in both genera (as well as many other species in Protopsyllidioidea). Thirdly, available records of *C. incerta* and *C. issykkulica* do not provide any data on preserved body structures (aside from wings) that can be compared with *Poljanka*. Therefore, the traits [swollen apical flagellomere] and [first tarsomere longer than second tarsomere] used by [18] to help assign *Cicadellopsis hirsuta* **comb. nov.** to *Poljanka* (rather than *Cicadellopsis*) cannot be confidently relied upon as their status is truly unknown in *Cicadellopsis*. Finally, we note that *Cicadellopsis hirsuta* **comb. nov.** possesses a branched vein RA in the forewing, as observed in the present material.

The distinctive characters between the two genera, as proposed by [24] (p. 39) and [18] (p. 41, remarks section), can be narrowed down to the branching (or not) of RA. Based on the available material and consequent data, *Cicadellopsis hirsuta* **comb. nov.** has vein RA forked, and thus, a more reasonable placement for the species would be in *Cicadellopsis*. The taxon also conforms to the diagnosis of *Cicadellopsis* proposed by [9] based on forewing diagnostic characters: (1) costal field widened near the middle, (2) stem of R very short, and (3) vein RA_1_ weak. Nonetheless, a careful and comprehensive re-examination of all specimens assigned to *Cicadellopsis* and *Poljanka* is necessary, particularly individuals with more complete body structures. The status of vein RA_1_ is variable in the preserved specimens of *Cicadellopsis hirsuta* **comb. nov.**, which casts doubt on the reliability of this trait as a (sole) diagnostic character for distinguishing between *Cicadellopsis* and *Poljanka*. Consequently, we cannot rule out the possibility that a branched RA might be present in some of the species assigned to *Poljanka*, especially since our knowledge comes mostly from very limited and often incompletely preserved individuals. A thorough revision, combined with the discovery of new material, would help to identify the true diagnostic characters (if any) that support a distinction between the two genera.

*Cicadellopsis hirsuta* **comb. nov.** has a very similar forewing venation pattern to *Cicadellopsis incerta*. Some small differences can be detected between the two species, i.e., vein Sc (absent in *C. hirsuta* while present and free in membrane in *C. incerta*), branching position of RA (at wing mid-length in *C. hirsuta* while after wing mid-length in *C. incerta*), length of pterostigma cell (length to width ratio of 3.5:1 in *C. hirsuta* while 2.5:1 in *C. incerta*), and shape of vein RP (sharply curved at apex in *C. hirsuta* while straight in *C. incerta*) [9]. *Cicadellopsis hirsuta* **comb. nov.** differs from *Cicadellopsis issykkulica* by the longer wing (about 1.5–1.75 times the length of holotype of *C. issykkulica*), slightly smaller forewing ratio (2.4:1 in *C. hirsuta* while 2.7:1 *C. issykkulica*), and the shape of the CuA cell (CuA_1_ less curved in *C. hirsuta*) [9].

It is noteworthy that *Cicadellopsis hirsuta* **comb. nov.** possesses a hind wing with an unbranched vein M, and a wide fork CuA, which are traits also described in *Poljanka kukalovae*. These hind wing features were initially considered diagnostic characters of *Cicadellopsis* by [9] before the establishment of *Poljanka*. Nonetheless, they are very general and likely found in both genera. In fact, the general venation pattern is very close to that of *Tripsyllidium wadei* Evans, 1956 (including the presence of a common stem R + M) [23], thus supporting the idea that the type material of *T. wadei* is a protopsyllidiid hind wing as suggested by [9,23].

None of the available specimens of *Poljanka jiyuanensis* **sp. nov.** display a clear distal branching or obtuse angular-like bending of RA; consequently, we opt to assign the new species to *Poljanka* for the time being. *Poljanka jiyuanensis* **sp. nov.** differs from the other species assigned to *Poljanka* by several characters of the forewing (Table 2), e.g., the shape and size of the wing, the shape of the CuA cell, and the lengths of stem R and vein CuA_2_. The type material of *Poljanka shurabensis* is poorly preserved, with only a fragment of the forewing described and illustrated in [9]; thus, it is difficult to compare with the new taxon. Nonetheless, the forewing of *Poljanka shurabensis* is stated to be around 6.5 mm in length (preserved length of 5.5 mm) while the forewing of *Poljanka jiyuanensis* **sp. nov.** ranges between 2.4 and 2.9 mm.

A biostratigraphic correlation was proposed between the Yangshuzhuang Formation of Henan Province and the Daohugou beds of Haifanggou Formation at Inner Mongolia—both believed to be Middle to earliest Late Jurassic in age—based on the presence of a *Triglypta-Qaidamestheria* type conchostracan assemblage [6,41]. According to the recent International Chronostratigraphic Chart published by the International Commission on Stratigraphy in September, 2025, the boundary between the Middle and Late Jurassic is set at 161.5 ± 1.0 Ma. On this basis, the Yangshuzhuang Formation and Haifanggou Formation are considered to be of late Middle Jurassic in age. Above these fossiliferous horizons, the boundary with the overlying Ma’ao/Tiaojishan formation is represented by an unconformity (refer to Yanshan Movement Phase A2 [41]) that is also correlated. More evidence of biostratigraphic correlation comes from fossil insects. Two insect species, the hemipteran *Jurocercopis grandis* Wang and Zhang, 2009 [33,45] and the trichocerid *Eotrichocera (Archaeotrichocera) ephemera* Zhang, 2006 [35,46], have been reported from both the Haifanggou Formation (Daohugou beds) and the Yangshuzhuang Formation. *Cicadellopsis hirsuta* commonly occurs in the uppermost fossil layers of the Daohugou beds, and is also concentrated in the upper part of the Yangshuzhuang Formation. Moreover, a new species of *Proptychopterina* (Diptera) has been described from the Yangshuzhuang Formation and resembles *P*. *opinata* from Daohugou [40]. The co-occurrence of *Cicadellopsis hirsuta* **comb. nov.**, showcased herein, is a new example of the entomofaunal similarities between the two localities. It is also new evidence indicating that the early assemblage of the Yanliao biota, represented by the Daohugou biota, was distributed at least as far south as Central China.

## 5. Conclusions

Protopsyllidioids are relatively common in Mesozoic strata of China. Exploring their biodiversity is crucial for establishing a comprehensive understanding of the superfamily’s evolutionary history and, by extension, the relationships between the basal Sternorrhynchan lineages. A careful re-examination of the material belonging to species of *Poljanka* and *Cicadellopsis* is required in order to clearly identify distinctive characters of the wings and other body structures between the two genera and hence support their separation.

## Figures and Tables

**Table 2:** Comparative table detailing forewing characters of species assigned to *Poljanka.* (Abbreviations: L, length; W, width).

Species	Length	L/W Ratio	Stem R Compared to Stem M + CuA	Shape of RA	Fork M Compared to Stem M	CuA Cell L/W Ratio	Length of CuA_2_
*P. curticapillata*	3.8–4.2 mm	1.8:1	Longer	Straight	Longer	2.45:1	0.31 × length of CuA_1_
*P. jiyuanensis* **sp. nov**	2.4–2.9 mm	1.8–2.0:1	Shorter	Mostly straight, weakly curved distally	Longer	3.5:1	0.55 × length of CuA_1_
*P. kukalovae*	3 mm	2.55:1	?	Curved towards anterior wing margin	Longer	?	?
*P. sharovi*	2.8 mm	2.6:1	Equal or slightly longer	Weakly sinusoidal	Equal	6.3:1	0.44 × length of CuA_1_
*P. shurabensis*	5.5 mm preserved	?	?	Mostly straight, curved near apex	Longer	?	?
*P. strigosa*	3.8–4.2 mm	2.2–2.7:1	Equal	Basally straight with strong arch near apex	Longer	3.8:1	0.15 × length of CuA_1_
*P. ventriculosa*	4.0–4.2 mm	2.3–2.4:1	Slightly shorter?	Mostly straight, weakly curved near apex	Longer	4:1	0.46 × length of CuA_1_

## Data Availability

The original contributions presented in this study are contained within the article. Further inquiries can be directed to the corresponding author.
